# Short-Term Effects of Three Types of Hamstring Stretching on Length, Neurodynamic Response, and Perceived Sense of Effort—A Randomised Cross-Over Trial

**DOI:** 10.3390/life12101666

**Published:** 2022-10-21

**Authors:** Carlos López-de-Celis, Pedro Izquierdo-Nebreda, Vanessa González-Rueda, Aïda Cadellans-Arróniz, Jacobo Rodríguez-Sanz, Elena Bueno-Gracia, Albert Pérez-Bellmunt

**Affiliations:** 1 Faculty of Medicine and Health Sciences, Universitat International de Catalunya, 08195 Barcelona, Spain; 2 ACTIUM Anatomy Group, Universitat Internacional de Catalunya, 08195 Barcelona, Spain; 3 Fundació Institut Universitari per a la Recerca a l’Atenció Primària de Salut Jordi Gol i Gurina, 08007 Barcelona, Spain; 4 Faculty of Health Sciences, Universidad de Zaragoza, 50009 Zaragoza, Spain

**Keywords:** flexibility, hamstrings, neurodynamics, stretching

## Abstract

Background: Stretching techniques for hamstring muscles have been described both to increase muscle length and to evaluate nerve mechanosensitivity. Aim: We sought to evaluate the short-term effects of three types of hamstring stretching on hamstring length and report the type of response (neural or muscular) produced by ankle dorsiflexion and perceived sense of effort in asymptomatic subjects. Methods: A randomised cross-over clinical trial was conducted. A total of 35 subjects were recruited (15 women, 20 men; mean age 24.60 ± 6.49 years). Straight leg raises (SLR), passive knee extensions (PKE), and maximal hip flexion (MHF) were performed on dominant and non-dominant limbs. In addition, the intensity of the applied force, the type and location of the response to structural differentiation, and the perceived sensation of effort were assessed. Results: All stretching techniques increased hamstring length with no differences between limbs in the time*stretch interaction (*p* < 0.05). The perceived sensation of effort was similar between all types of stretching except MHF between limbs (*p* = 0.047). The type of response was mostly musculoskeletal for MHF and the area of more neural response was the posterior knee with SLR stretch. Conclusions: All stretching techniques increased hamstring length. The highest percentage of neural responses was observed in the SLR stretching, which produced a greater increase in overall flexibility.

## 1. Introduction

Muscle stretching aims to correct soft tissue tightness [1] and prevent injuries and dysfunctions related to a loss in muscle length [2,3,4,5]. Hamstrings are a muscle group for which a variety of type of stretches have been described [6,7,8,9]. The straight leg raise (SLR) [10,11], the passive knee extension (PKE) [12,13], and the maximum hip flexion (MHF) [14] are the most common stretching techniques used for hamstring muscles. These three techniques use different sequences of movements to reach the final position, where subjects commonly report tension. The SLR starts with full knee extension and performs hip flexion [15], the PKE starts from 90° of hip flexion and performs passive knee extension [13], and the MHF starts in submaximal hip flexion and performs knee extension [14]. Some of these stretching techniques are described both to increase muscle length [16,17] and to evaluate lumbar nerve roots and sciatic nerve mechanosensitivity [18,19]. This raises the question of the specificity of the techniques and their effects on nerve or muscular structures. There is one study that observed that changing the ulnar nerve stretch sequence also changed the ulnar nerve tension at the elbow [20]. For that reason, it is possible that different sequences of hamstring/sciatic nerve stretches will generate different responses.

In clinical practice, a structural differentiation manoeuvre is performed to discriminate whether the tension produced during a neurodynamic test arises from muscle or neural tissue [21,22] This manoeuvre can be head flexion, internal rotation, and/or adduction of the hip or dorsiflexion of the ankle. This manoeuvre will depend on the location of the symptomatology. The structural differentiation is a manoeuvre able to modify the strain in the nervous system without generating any change in adjacent muscular structures [15,23]. This phenomena is related to the anatomical continuous nature of the nervous system, which allows the tension generated at a point of the system to transmit to great distances to more distant areas [24]. Ankle dorsiflexion used as structural differentiation has been shown (in cadaveric studies) to produce changes in the strain and excursion of the sciatic nerve at the upper thigh and tibial nerve at the posterior knee at different degrees of hip flexion during the SLR [22,25]. In contrast, ankle movement did not affect the biceps femoris muscle in some locations. These findings supported the use of ankle dorsiflexion as structural differentiation manoeuvre in certain circumstances.

Tissue-directed stretching interventions can preferentially load muscular or non-muscular structures such as peripheral nerves. It has been shown that both nerve-directed and muscle-directed interventions in the calf region are effective to increase the range of motion in healthy subjects [26]. However, it has also been shown that nerve-directed stretching techniques induce chronic changes in the mechanical properties of the nerve but not of the muscle. The same is true when techniques are directed at the muscle, where only the muscle shows sustained changes but not the nerve. Thus, it is possible to target and induce changes in the mechanical properties of nerves or muscular structures by means of specific stretching techniques [26]. 

We hypothesised that there would be a short-term difference in hamstring length between the three hamstring stretching typologies (SLR, PKE, and MHF) and that there would be differences in the type of response (neural or muscular) produced by ankle dorsiflexion and the perceived sensation of strain.

The study aims were to evaluate the short-term effects on hamstring length of three types of hamstring stretching (SLR, PKE, and MHF) and to report the type of response (neural or muscular) produced by the ankle dorsiflexion as the perceived sense of effort in asymptomatic subjects.

## 2. Materials and Methods

### 2.1. Study Design

A randomised cross-over clinical trial was carried out between December 2020 and February 2021. The study was registered on ClinicalTrials.gov (NCT04763798) and was approved by a local ethics committee of Universitat Internacional de Catalunya (CBAS-2019-12). The procedures followed were in accordance with the Declaration of Helsinki (revised Fortaleza 2013). The CONSORT statement guidelines were used to prepare the study.

### 2.2. Sample

The sample was composed of a total of 35 asymptomatic volunteers (15 women, 20 men) with a mean age of 24.60 years (SD = 6.49) from the Faculty of Medicine and Health Sciences for Universitat Internacional de Catalunya. The inclusion criteria were as follows: subjects over 18 years old who signed the informed consent. The exclusion criteria were as follows: (1) injury in the thigh 6 months previously, (2) self-reported information of any orthopaedic problem or surgery in the lower limbs, (3) back pain or previous surgery in the spine, or (4) systemic or neurological disorders.

The sample size was calculated based on previous pilot study using the GRANMO 7.12 program. A two-sided test analysis (two paired means) assuming an α risk of 0.05 and a β risk of 0.20 (i.e., 80% power) was performed for an expected improvement of 5.6 cm (SD 10.56 cm) on the Modified back-saver sit-and-reach test. Assuming an estimated dropout rate of 20%, 35 participants were required. The pilot study sample comprised 10 subjects (6 men and 4 women). The stretching and assessment procedure was the same as the one performed later in the study.

### 2.3. Intervention

**Figure 1:**
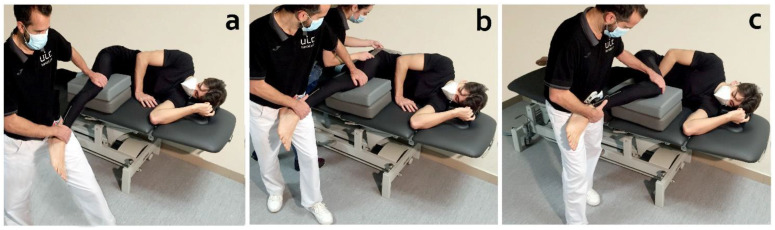
Stretch position for (**a**) straight leg raise, (**b**) passive knee extension, and (**c**) maximum hip flexion.

**Figure 2:**
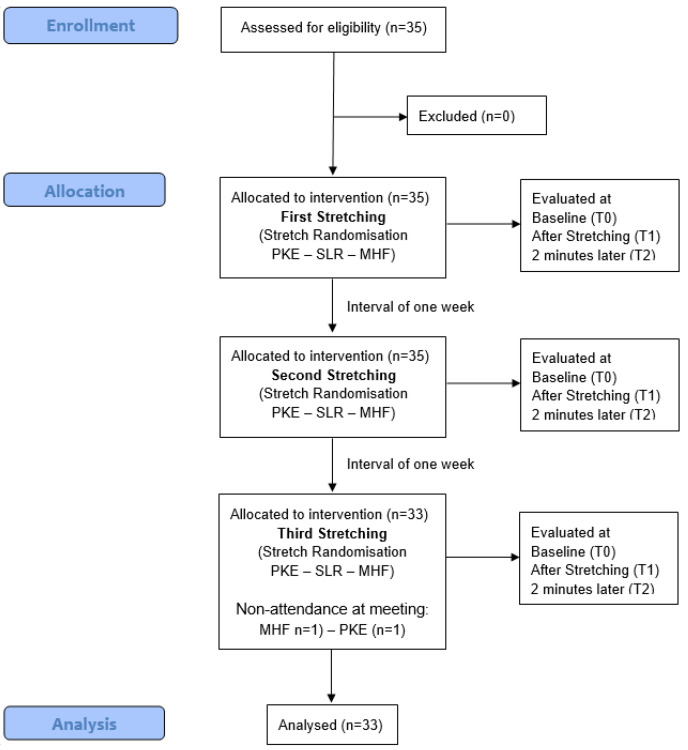
Consolidated Standards of Reporting Trial (CONSORT) flow diagram.

All subjects received the three stretching techniques in both limbs (dominant and non-dominant limb) (Figure 1). Interventions were performed on three different days and only one of the three stretching techniques was performed in each session. The rest period between sessions was one week. Stretching techniques were performed in a random order (Figure 2). A random-number generator (random.org) was used for randomization. The order of application of the techniques was placed in a concealed opaque envelope and the order of application to each participant was randomly assigned. 

### 2.4. Outcome Measures

Hamstring length was assessed as the main outcome using the modified back-saver sit-and-reach test [27] with a blinded evaluator. In addition, structural differentiation response (passive ankle dorsiflexion), strength application during the stretching technique (dynamometer) [28], and perceived sensation of effort at the end of the stretching (Borg scale) [29] were evaluated at baseline (T0), after the application of each type of stretching (T1), and 2 min later (T2).

**Figure 3:**
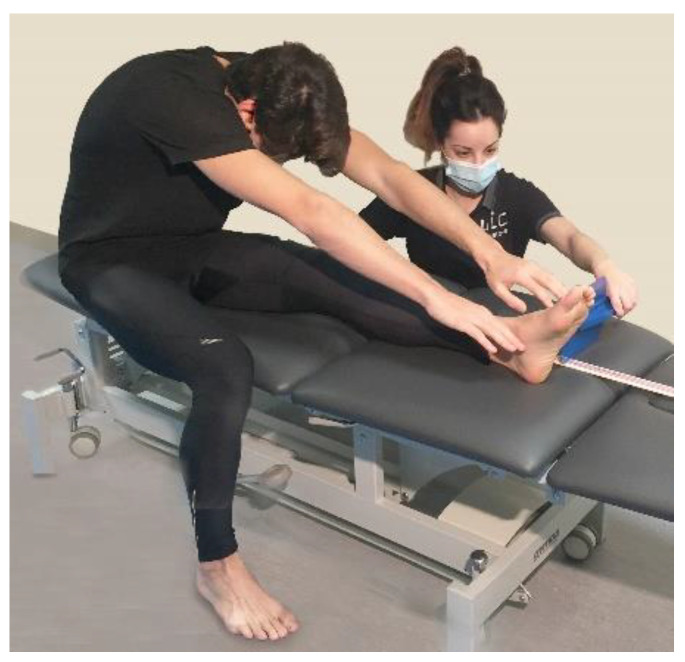
Modified back-saver sit-and-reach test.

In the modified back-saver sit-and-reach test, subjects performed a single-leg sit-and-reach on a bench. The ankle position of the assessed leg was left free without conditioning any dorsal flexion. The untested leg was placed on the floor with the knee at 90° approximately. A measuring tape was placed on the bench. Subjects aligned the sole of the foot of the tested leg with the 100 cm mark on the measuring tape. Thereafter, subjects were asked to reach forward as far as possible while maintaining the knees, arms, and fingers fully extended and keep their two hands next to each other with the palms down as shown in Figure 3. The score was recorded as the most distant point on the bench [27,30].

Structural differentiation response: Passive ankle dorsiflexion was performed as a structural differentiation manoeuvre in the final position of all types of stretching. Subjects were asked to indicate if the sensation during the stretching was modified during the ankle dorsiflexion (neural response) or was the same (musculoskeletal response) and the results were registered. The type and location of response referred during the stretching were also registered.

**Figure 4:**
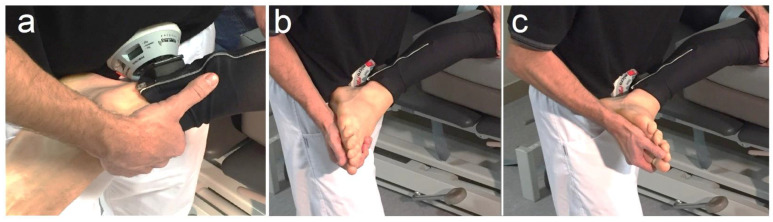
(**a**) Position of the dynamometer for force measurement; (**b**) initial ankle dorsiflexion position (structural differentiation manoeuvre); (**c**) final ankle dorsiflexion position (structural differentiation manoeuvre).

Strength applied: The intensity of the force applied during each type of stretching was measured using a manual dynamometer (Micro-FET2, Hoggan Scientific, Salt Lake City, UT, USA) in Newtons. The dynamometer was applied on the distal leg proximal to the ankle joint. The physiotherapist performed the stretching until a marked resistance (end field) was felt and then the force was registered (Figure 4a).

Perceived sensation of effort: After the stretching application, all subjects were asked about their perceived sensation of effort. They were asked about the location and intensity of the tension sensation during the technique. A numeric scale ranging from 0 (“no tension”) to 10 (“worst tension imaginable”) was used to indicate the intensity of tension sensation during the stretching technique. A body chart depicting the right and left lower extremity and divided into 5 areas (calf, posterior knee, posterior thigh, buttock, and other) was used to document the distribution of the sensation during the stretching; each individual was asked to mark the location of the perceived responses.

### 2.5. Procedure

All stretching techniques were performed with the subjects in lateral decubitus on a bench to avoid the influence of the lower limb’s weight in determining the stretch’s end field. The stretched lower limb was on a movable support. If necessary, the height of the mobile support was adjusted to avoid changes in abduction or adduction that could influence the measurements (Figure 1). The knee was kept in full extension for SLR and then hip flexion was performed until a notice marked resistance (end field). For the PKE, the hip was kept in 90° flexion and then knee extension was performed. For the MHF, a maximal hip flexion was performed and then knee extension was applied. A universal goniometer was used to measure the hip flexion angle according to standard goniometry guidelines [31]. All types of stretching were applied by a physical therapist with more than 15 years of experience until the end field or until the point of maximal tolerance referred by subjects. At the end of the stretching, the structural differentiation manoeuvre (ankle dorsiflexion) was performed and the type of response (musculoskeletal or neural) was registered (Figure 4b,c).

The stretches lasted 30 s followed by a 15 s rest period; the procedure was repeated five times. All stretching techniques were performed by the same physiotherapist first on one limb and then on the other according to the randomisation order. A second physical therapist assisted in the manual stabilization of the subjects during the technique, controlled the duration of the stretching and the resting time, and registered the response to structural differentiation and the perceived sensation of effort of subjects during the application of the technique. Another blind assessor evaluated the modified back-saver sit-and-reach test and the perceived sensation of effort for subjects during the application of the technique.

### 2.6. Statistical Analysis

For statistical analysis, IBM SPSS Statistics for Windows, version 20.0 (Armonk, NY, USA: IBM Corp) was used to assess differences in the hamstring length, type of response, intensity of applied force, and perceived sensation of effort for each time interval and with each stretch performed. 

A descriptive analysis was carried out. The mean and standard deviation were calculated for quantitative variables. Frequencies were calculated for demographic qualitative variables. The Shapiro–Wilk test was used to determine non-normal distribution of quantitative data.

This linear mixed model was performed for the hamstring-length dependent variable in which the type of stretch was the between-subjects factor and time was the within-subjects factor. If the assumption of sphericity was violated, the Greenhouse–Geisser correction was utilized for interpretation. When a statistically significant effect was noted, a post hoc analysis was performed and the Bonferroni correction was used to adjust for multiple comparisons. 

A Wilcoxon test or paired *t*-test was used to compare the intensity of the applied force and the perceived sensation of effort between the limbs and the stretch.

A Mann–Whitney U-test or independent *t*-test was used to compare the intensity of force applied and perceived sensation of effort between stretches. 

The effect sizes were calculated using Cohen’s d coefficient [32]. An effect size >0.8 was considered large; around 0.5, intermediate; and <0.2, small.

For the variable type of response and the perceived response’s location, a frequency analysis and a comparative analysis between stretching types was conducted using chi-squared and Fisher’s exact statistics, respectively. The level of significance was set at *p* < 0.05.

## 3. Results

The demographic characteristics of the sample are shown in Table 1. Two subjects dropped out of the study during stretching sessions (for non-attendance of the meeting). 

### 3.1. Hamstring Length

In dominant limbs, there were statistically significant main effects in specific hamstring length for time (F = 49.652 (*p* < 0.001)) but not for stretch (F = 0.382 (*p* = 0.684)). There was no significant interaction between time and stretch (F = 1.759 (*p* = 0.141)).

In non-dominant limbs, there were significant main effects in specific hamstring length for time (F = 77.394 (*p* < 0.001)) but not for stretch (F = 0.283 (*p* = 0.718)). There was no significant interaction between time and stretch (F = 1.090 (*p* = 0.358)).

Table 2 provides the results before and after each stretching session and Table 3 shows the between-limbs differences. 

In the analysis according to gender, women had statistically significant main effects in specific hamstring length for time (F = 46.768 (*p* < 0.001)) but not for dominant (F = 0.154 (*p* = 0.701)) and not for stretch (F = 0.865 (*p* = 0.390)). There was no significant interaction between time and stretch (F = 1.526 (*p* = 0.207)). 

In the analysis according to gender, men had statistically significant main effects in specific hamstring length for time (F = 15.587 (*p* < 0.001)) but not for dominant (F = 1.418 (*p* = 0.248)) and not for stretch F = 0.725 (*p* = 0.456). There was no significant interaction between time and stretch (F = 0.722 (*p* = 0.448)).

Table 4 shows the results before and after each stretching session according to dominance and gender. Table 5 shows the differences between the different moments of each stretching session according to dominance and gender.

### 3.2. Intensity of Applied Force

There were no statistically significant differences in the intensity of force applied between limbs (*p* > 0.05) (Table 6). However, there was a statistically significant difference in the intensity of the force applied between type of stretching. There was a statistically significant difference in intensity of force applied between PKE and SLR stretching of both dominant (27.1 N ± 38.8; *p* = 0.001) and non-dominant limbs (26.2 N ± 29.7; *p* < 0.001) and between PKE and MHF stretching of dominant limbs (20.8 N ± 37.3; *p* = 0.003).

There was no statistically significant difference between limbs according to dominance in intensity force according to gender. According to dominance, there was no statistically significant difference in force intensity between genders (Table 7).

### 3.3. Perceived Sensation of Effort

There was only a minimal statistically significant difference in the perceived sensation of stretching with the Borg scale between limbs with the MHF stretching (0.5 points; *p* = 0.047), as well as between the perceived sensation of effort between PKE and SLR stretching, both dominant (1.5 points; *p* = 0.022) and non-dominant (1.4 points; *p* = 0.005).

Only SLR stretching achieved a statistically significant difference among the limbs according to dominance in perceived sensation by gender (*p* = 0.033). The PKE stretching achieved a statistically significant difference in perceived sensation according to gender in the dominant (*p* = 0.011) and non-dominant limbs (*p* = 0.008), with a greater perceived sensation exertion in males. Furthermore, in the SLR stretching, a statistically significant difference was reached in the non-dominant limbs, with a greater perceived sensation exertion in men (*p* = 0.007) (Table 7).

### 3.4. Type and Location of Responses

Data on the type of response (musculoskeletal or neural) to structural differentiation and its location are shown in Table 8.

The stretching with the highest musculoskeletal response was the MHF with 66.7% in dominant limbs and 78.8% in non-dominant limbs. The stretch type with the highest neural response was SLR with 75.8% in dominant limbs and 72.7% in non-dominant limbs. Regarding the response zone, we found a statistically significant difference between the zones between PKE and SLR stretching of dominant limbs (*p* = 0.014) as well as between dominant and non-dominant limbs in each type of stretch (PKE *p* < 0.001, SLR *p* < 0.001, MHF *p* = 0.046). The area where the highest neural response occurred in both limbs was in the posterior knee: 36.4% in dominant limbs and 33.3% in non-dominant limbs Table 8.

In the analysis according to gender, the behaviour was found to be similar. No statistically significant differences were found in the different types of stretching according to dominance (dominant: PKE *p* = 0.417, SLR *p* = 0.108, MHF *p* = 0.222; non-dominant: PKE *p* = 0.465, SLR *p* = 0.308, MHF *p* = 0.344) (Table 9).

## 4. Discussion

The similarities between the stretching techniques of the hamstring muscles and some neurodynamic tests such as the SLR test raise a question regarding the specificity of these techniques for muscle tissue and their physiological effects. This study aimed to evaluate the short-term effects on hamstring length of three types of stretching (SLR, PKE, and MHF) and report the type of response (neural or muscular) produced by ankle dorsiflexion and perceived sense of effort in asymptomatic subjects. The results showed that SLR stretching produced more neural responses followed by PKE. SLR stretches also produced a greater increase in hamstring length as indicated by an increased score on the modified back-saver sit-and-reach test. In all types of stretching, the end field was reached with application of a similar force intensity except for the force applied in PKE and SLR stretching in both dominant and non-dominant limbs and between PKE and MHF stretching in dominant limbs. PKE stretching required a minor force to reach the end field. Almost all of the effect sizes were intermediate, which was expected for 5 min of stretching.

The SLR is an evolution of the Lassegue test, which was designed to examine mechanosensitivity and mechanical function impairment of the nervous system [19,21,33,34,35,36]. The SLR showed increased tension in neural structures both in vivo and in vitro [37,38,39,40]. In addition, ankle dorsiflexion has been shown to increase strain and produce distal excursion of the sciatic nerve without affecting muscle structures such as the biceps femoris muscle [22,25]. Due to the differential behaviour observed between the nerve and the muscle at the posterior thigh, ankle dorsiflexion has been proposed as an excellent manoeuvre for a differential diagnosis of posterior thigh pain [22,25]. In the present study, 77.1% of the subjects showed a neural response during ankle dorsiflexion in the SLR stretching. SLR has a similar mechanism of execution to the neurodynamic test. Based on the existing literature, this could mean that this type of stretching produced more neural tension than muscular. Furthermore, most subjects placed the effort sensation perceived for stretching in the popliteal fossa during this type of stretching. This finding was in line with previous studies on the usual response to the SLR test in healthy subjects, in which the most frequently reported response was tension in the posterior knee [19,21,33,41,42,43].

MHF stretching obtained the lowest number of neural responses. This fact suggested that the MHF technique could be more specific to muscle tissue than the other techniques analysed, which could be due to several factors. On the one hand, greater stability of the pelvis achieved by stabilising the contralateral hip with a strap could minimise compensatory movements at the lumbar level during stretching [14,44], thus helping to direct the tension on the muscle tissue in a more specific way. The other factor that could explain the greater specificity of this stretch is that hip flexion has a more significant effect on increasing muscle tension than neural tension. Thus, although both the hamstring muscles and the sciatic nerve increase their tension during hip flexion [38,40], muscles may do it to a greater extent. Finally, PKE stretching was shown to affect the musculature only in 42.4% of the cases, which was an intermediate value compared to those of the other two stretching techniques analysed.

All types of stretching improved flexibility at the end of the stretch and 2 min later; we found no statistically significant differences between type of stretching in the time*stretch interaction. SLR stretching achieved the most notable gain, with MHF showing similar results in the dominant limbs. In the non-dominant limbs, PKE obtained values close to those of the SLR.

In this study, only three modalities of stretching were evaluated; however, in muscle stretching, there were various parameters that included intensity, position, frequency, and typology (ballistic, PNF, and static active or static passive). The combination of these parameters has been shown to act on the anatomical and physiological properties of the muscle. It was found that length gains seemed more linked to the frequency of stretching than to the time per session [45] Our study referred to very short-term effects. A recent study showed that both nerve- and muscle-directed types of stretching could increase the range of motion in the calf muscles [26]. However, this increase in the range of motion was achieved through different physiological mechanisms. While nerve-directed stretching techniques significantly decreased sciatic shear wave velocity (an estimate of stiffness), the muscle-directed group exhibited an overall reduction in the plantar flexor’s shear wave velocity. A study by Andrade et al. (2020) was the first to show that it was possible to reduce nerve stiffness and improve joint range of motion. In clinical practice, these nerve-directed stretching techniques could be particularly beneficial in those disorders in which increased local nerve stiffness in conjunction with decreased maximal joint range of motion are present, such as carpal tunnel syndrome or lumbar radiculopathy [26,46,47,48,49]. Similarly, it may be of interest to decrease neural tension in patients with sciatic nerve irritation. In that case, the application of MHF stretching would be the best option. Most previous studies based the stretching intensity on the subjects’ perception or discomfort, so tolerance to stretching could increase the gain [45]; however, Hoge KM. et al. found that women had an increased tolerance but no change in muscle stiffness after a passive stretching protocol [50] In our comparative analysis according to gender, we found no relevant differences. The results were similar to what we found in our previous study in which women reported lower scores in perceived sensation compared to men. However, there were no relevant differences in stretch gain or neural response zones.

Based on the data provided in this study and by following the line of studies by Bueno-Gracia E. et al. [22,25], we believe that it could be interesting to determine what degree of tension is reached in the nerve and muscle tissue in vitro when using the types of stretching performed in this study.

### Study Limitations

This study only analysed three types of stretching, so we cannot be sure if other types of stretching can produce different results. In addition, we did not have a control group in which no stretching is applied, thus the real difference compared to no intervention for this type of stretching is unknown. Likewise, the population included was young and different results in other age ranges could be reported. In addition, a shortening variable was not established as an inclusion criteria, thus increases in hamstring length could have been different between subjects.

## 5. Conclusions

All of the stretching techniques increased the hamstring length. The highest percentage of neural responses was observed for SLR stretching followed by PKE. This could suggest a possible effect of these techniques on nerve rather than muscle tissue. The straight leg raise stretching produced a greater increase in overall flexibility. The intensity of force applied in the stretches to reach the end field was similar except for PKE with dominant and non-dominant SLR and PKE and MHF. The perceived sensation of effort was similar between all types of stretching. The type of response was mainly musculoskeletal for MHF while the area with a greater neural response was the posterior knee with the SLR stretch.

## Figures and Tables

**Table 1:** Subjects’ demographic characteristics.

	Mean ± SD or n (%)
Age (year)	24.60 ± 6.49
Gender	
Men	20 (57.1%)
Women	15 (42.9%)
Dominance	
Right	27 (77.1%)
Left	8 (22.9%)

Abbreviations: SD, standard deviation; n, number.

**Table 2:** Outcomes of the modified back-saver sit-and reach test.

	T0	T1	Difference T0–T1				T2	Difference T0–T2			
Variables	Mean ± SD	Mean ± SD	Mean	95% CI	*p*	ES	Mean ± SD	Mean	95% CI	*p*	ES
Dominant Limbs											
PKE—Hamstring length (cm)	−0.4 ± 10.0	2.4 ± 10.1	2.8	[1.217; 4.358]	0.000	0.28	2.3 ± 10.3	2.7	[1.386; 4.008]	0.000	0.27
SLR—Hamstring length (cm)	−1.8 ± 11.1	2.3 ± 10.5	4.1	[2.579; 5.652]	0.000	0.38	2.4 ± 11.6	4.2	[2.506; 5.979]	0.000	0.37
MHF—Hamstring length (cm)	−0.8 ± 10.4	2.6 ± 11.1	3.3	[1.654; 4.994]	0.000	0.32	3.5 ± 11.1	4.2	[2.261; 6.200]	0.000	0.40
Non-Dominant Limbs											
PKE—Hamstring length (cm)	−0.9 ± 11.5	1.4 ± 11.2	2.3	[0.726; 3.971]	0.003	0.28	3.1 ± 11.0	4.0	[2.539; 5.461]	0.000	0.36
SLR—Hamstring length (cm)	−1.8 ± 11.5	1.2 ± 12.1	3.1	[1.862; 4.338]	0.000	0.25	2.7 ± 11.4	4.6	[3.187; 5.922]	0.000	0.39
MHF—Hamstring length (cm)	−0.5 ± 10.0	1.2 ± 10.9	1.7	[0.363; 3.061]	0.009	0.16	2.7 ± 11.2	3.2	[1.475; 4.949]	0.000	0.30

Abbreviations: cm, centimeter; CI, confidence interval; PKE, passive knee extension; SLR, straight-leg-raise; MHF, maximum hip flexion; SD, standard deviation; ES, effect size Cohen’s d.

**Table 3:** Inter-limb outcomes in the analysis of the modified back-saver sit-and reach test.

	Difference T0–T1			Difference T0–T2		
Variable	Dominant Limbs	Non-Dominant Limbs		Dominant Limbs	Non-Dominant Limbs	
	Mean ± SD	Mean ± SD	*p*	Mean ± SD	Mean ± SD	*p*
PKE—Hamstring length (cm)	2.8 ± 3.6	2.3 ± 3.7	0.509	2.7 ± 3.0	4.0 ± 3.3	0.096
SLR—Hamstring length (cm)	4.1 ± 3.5	3.1 ± 2.8	0.119	4.2 ± 3.9	4.6 ± 3.1	0.672
MHF—Hamstring length (cm)	3.3 ± 3.8	1.7 ± 3.1	0.046	4.2 ± 4.5	3.2 ± 3.9	0.341

Abbreviations: cm, centimeter; PKE, passive knee extension; SLR, straight-leg-raise; MHF, maximum hip flexion; SD, standard deviation.

**Table 4:** Outcomes of the modified back-saver sit-and reach test according to gender.

	T0	T1	Difference T0–T1				T2	Difference T0–T2			
Variables	Mean ± SD	Mean ± SD	Mean	95% CI	*p*	ES	Mean ± SD	Mean	95% CI	*p*	ES
Dominant Limbs											
Women											
PKE—Hamstring length (cm)	4.7 ± 8.3	6.3 ± 8.3	1.4	[−0.956; 3.802]	0.421	0.19	6.3 ± 8.9	1.4	[−0.340; 3.207]	0.136	0.19
SLR—Hamstring length (cm)	4.1 ± 11.5	8.4 ± 10.9	4.3	[1.698; 6.876]	0.001	0.38	9.0 ± 11.4	4.9	[2.460; 7.380]	0.000	0.43
MHF—Hamstring length (cm)	6.5 ± 11.1	8.9 ± 12.4	2.3	[0.625; 4.018]	0.007	0.20	9.9 ± 12.3	3.4	[1.096; 5.661]	0.004	0.29
Men											
PKE—Hamstring length (cm)	−4.3 ± 9.1	−0.5 ± 10.2	3.8	[1.413; 6.087]	0.001	0.39	−0.6 ± 10.2	3.7	[2.009; 5.291]	0.000	0.38
SLR—Hamstring length (cm)	−5.5 ± 10.4	−1.3 ± 9.6	4.2	[2.271; 6.129]	0.000	0.42	−1.4 ± 11.2	4.1	[1.659; 6.611]	0.001	0.38
MHF—Hamstring length (cm)	−4.5 ± 9.3	−0.4 ± 10.4	4.1	[1.616; 6.654]	0.001	0.42	0.4 ± 10.3	4.9	[2.042; 7.838]	0.001	0.50
Non-Dominant Limbs											
Women											
PKE—Hamstring length (cm)	4.3 ± 9.7	6.2 ± 9.7	2.4	[−0.110; 4.910]	0.063	0.20	7.7 ± 9.8	3.8	[1.542; 6.125]	0.001	0.35
SLR—Hamstring length (cm)	4.4 ± 10.0	8.1 ± 10.8	3.6	[1.242; 5.998]	0.003	0.36	9.4 ± 10.5	5.0	[2.815; 7.158]	0.000	0.49
MHF—Hamstring length (cm)	5.5 ± 11.0	7.4 ± 11.6	1.9	[0.294; 3.563]	0.019	0.17	8.6 ± 11.8	3.1	[1.640; 4.646]	0.000	0.27
Men											
PKE—Hamstring length (cm)	−4.6 ± 11.2	−1.9 ± 10.9	2.7	[0.369; 5.031]	0.020	0.24	−5.0 ± 22.4	−0.4	[−13.198; 12.408]	1.000	0.02
SLR—Hamstring length (cm)	−5.6 ± 11.4	−2.8 ± 11.8	2.7	[1.467; 3.983]	0.000	0.24	−1.0 ± 11.1	4.5	[2.668; 6.382]	0.000	0.41
MHF—Hamstring length (cm)	−3.3 ± 9.5	−1.7 ± 10.5	1.6	[−0.441; 3.591]	0.163	016	0.0 ± 11.2	3.3	[0.491; 6.109]	0.018	0.32

Abbreviations: cm, centimeter; CI, confidence interval; PKE, passive knee extension; SLR, straight-leg-raise; MHF, maximum hip flexion; SD, standard deviation; ES, effect size Cohen’s d.

**Table 5:** Outcomes of the dominance-modified sit and reach test according to gender.

	Difference T0–T1			Difference T0–T2		
Variable	Women	Men		Women	Men	
	Mean ± SD	Mean ± SD	*p*	Mean ± SD	Mean ± SD	*p*
Dominant Limbs						
PKE—Hamstring length (cm)	1.43 ± 2.06	3.75 ± 3.98	0.048	1.43 ± 2.53	3.65 ± 2.80	0.021
SLR—Hamstring length (cm)	4.29 ± 3.69	4.20 ± 3.29	0.942	4.92 ± 3.51	4.14 ± 4.22	0.563
MHF—Hamstring length (cm)	2.32 ± 2.42	4.14 ± 4.29	0.152	3.38 ± 3.25	4.94 ± 4.94	0.296
Non-Dominant Limbs						
PKE—Hamstring length (cm)	2.40 ± 3.58	2.70 ± 3.97	0.819	3.83 ± 3.27	−0.40 ± 21.81	0.463
SLR—Hamstring length (cm)	3.62 ± 3.39	2.73 ± 2.14	0.346	4.99 ± 3.09	4.53 ± 3.16	0.856
MHF—Hamstring length (cm)	1.93 ± 2.33	1.58 ± 3.43	0.734	3.14 ± 2.14	3.30 ± 4.79	0.907

Abbreviations: cm, centimeter; PKE, passive knee extension; SLR, straight-leg-raise; MHF, maximum hip flexion; SD, standard deviation.

**Table 6:** Inter-limb outcomes in the analysis of the intensity of applied force and perceived sensation.

	Dominant Limbs	Non-Dominant Limbs	
	Mean ± SD	Mean ± SD	*p*
Intensity of force (N)			
PKE	139.4 ± 25.3	147.3 ± 30.2	0.058
SLR	166.4 ± 34.2	173.5 ± 26.3	0.065
MHF	160.1 ± 23.4	167.0 ± 58.8	0.879
Perceived sensation (points)			
PKE	5.1 ± 3.1	5.2 ± 2.7	0.982
SLR	6.6 ± 2.0	6.6 ± 1.9	0.959
MHF	5.9 ± 2.3	6.4 ± 2.0	0.047

Abbreviations: N, Newtons; PKE, passive knee extension; SLR, straight-leg-raise; MHF, maximum hip flexion; SD, standard deviation.

**Table 7:** Outcomes of the analysis of the intensity of applied force and perceived sensation according to gender.

	Women	Men	
	Mean ± SD	Mean ± SD	*p*
Intensity of force (N)			
Dominant Limbs			
PKE	129.7 ± 28.8	145.0 ± 19.7	0.071
SLR	166.3 ± 23.9	164.9 ± 39.8	0.908
MHF	154.0 ± 19.4	162.2 ± 26.8	0.320
Non-Dominant Limbs			
PKE	139.9 ± 29.0	153.6 ± 29.1	0.176
SLR	169.7 ± 21.2	174.4 ± 29.7	0.612
MHF	147.5 ± 32.6	178.8 ± 68.5	0.057
Perceived sensation (points)			
Dominant Limbs			
PKE	3.8 ± 3.0	6.3 ± 2.8	0.011
SLR	6.2 ± 2.1	6.8 ± 1.8	0.393
MHF	4.9 ± 2.6	6.3 ± 1.9	0.80
Non-Dominant Limbs			
PKE	3.9 ± 2.7	6.3 ± 2.2	0.008
SLR	5.6 ± 1.9	7.3 ± 1.5	0.007
MHF	5.6 ± 2.6	6.8 ± 1.4	0.82

Abbreviations: N, Newtons; PKE, passive knee extension; SLR, straight-leg-raise; MHF, maximum hip flexion; SD, standard deviation.

**Table 8:** Type and location of responses during stretching performance.

	Musculoskeletal Response	Neural Response				
		Calf	Posterior Knee	Posterior Thigh	Buttock	Other
	n (%)	n (%)	n (%)	n (%)	n (%)	n (%)
Dominant						
PKE	14 (42.4%)	2 (6.1%)	13 (39.4%)	2 (6.1%)	-	2 (6.1%)
SLR	8 (24.2%)	3 (9.1%)	12 (36.4%)	10 (30.3%)	-	-
MHF	22 (66.7%)	1 (3.0%)	5 (15.2%)	4 (12.1%)	1 (3.0%)	-
Non-Dominant						
PKE	14 (42.4%)	1 (3.0%)	15 (45.5%)	2 (6.1%)	-	1 (3.0%)
SLR	9 (27.3%)	3 (9.1%)	11 (33.3%)	9 (27.3%)	1 (3.0%)	-
MHF	26 (78.8%)	-	5 (15.2%)	2 (6.1%)	-	-

Abbreviations: PKE, passive knee extension; SLR, straight-leg-raise; MHF, maximum hip flexion; n, number.

**Table 9:** Type and location of responses during stretching performance according to gender.

	Musculoskeletal Response	Neural Response				
		Calf	Posterior Knee	Posterior Thigh	Buttock	Other
	n (%)	n (%)	n (%)	n (%)	n (%)	n (%)
Dominant						
Women (n = 13)						
PKE	8 (61.5%)	1 (7.7%)	3 (23.1%)	-	-	1 (7.7%)
SLR	6 (46.2%)	-	4 (30.8%)	3 (23.1%)	-	-
MHF	9 (69.2%)	1 (7.7%)	3 (23.1%)	-	-	-
Men (n = 20)						
PKE	6 (30.0%)	1 (5.0%)	10 (50.0%)	2 (10.0%)		1 (5%)
SLR	2 (10.0%)	3 (15.0%)	8 (40.0%)	7 (35.0%)		-
MHF	13 (65.0%)	-	2 (10.0%)	4 (20.0%)	1 (5.0%)	-
Non-Dominant						
Women (n = 13)						
PKE	8 (61.5%)		4 (30.8%)	1 (7.7%)	-	-
SLR	5 (38.5%)	1 (7.7%)	4 (30.0%)	2 (15.4%)	1 (7.7%)	-
MHF	11 (84.6%)	-	2 (15.4%)	-	-	-
Men (n = 20)						
PKE	6 (30.0%)	1 (5.0%)	11 (55.0%)	1 (5.0%)	-	1 (5.0%)
SLR	3 (15.0%)	2 (10.0%)	8 (40.0%)	7 (35.0%)	-	-
MHF	16 (80.0%)	-	2 (10.0%)	2 (10.0%)	-	-

Abbreviations: PKE, passive knee extension; SLR, straight-leg-raise; MHF, maximum hip flexion; n, number.

## Data Availability

The datasets analysed during the current study are available from the corresponding author upon reasonable request. All data analysed during this study are included in this published article.
